# Proceedings of the Third Annual Meeting of the Michigan Dental Association

**Published:** 1858-06

**Authors:** 


### Proceedings of Societies.

---

### PROCEEDINGS OF THE THIRD ANNUAL MEETING OF THE MICHIGAN DENTAL ASSOCIATION.

The fourth meeting (third annual) of this association met at the office of Dr. Porter, in the city of Ann Arbor, on Monday the 11th of January, 1858, at 7 o'clock, P. M. There not being a sufficient number present for the transaction of business, the evening was passed in social intercourse.

Tuesday morning, January 12th. The association was called to order at 9½ o'clock, President Mansfield in the chair.

The minutes of the last annual and semi-annual meetings were read and approved.

The secretary then read communications from Drs. Taft and Watt, Cincinnati, Ohio; Messrs. Jones, White and McCurdy, New York; Dr. E. D. Ingersoll, Monroe, Michigan; Dr. L. T. Dryer, Constantine, Michigan, and Dr. G. W. Stone, Albion, Michigan;—which, on motion of Dr. Foster, were ordered to be placed on file and the thanks of the Association tendered to the gentlemen who had in this manner responded to the invitation to attend this meeting.

On motion, Dr. Bancroft was appointed a committee to correspond with Dr. Ingersoll and inform him that his resignation is *not accepted by the Association.*

[The following is that portion of Dr. Ingersoll's letter which calls for a resignation: "I am pleased to know that your circulars are again calling together my professional brethren, and were it not for the delicate state of my health, I should be very happy to respond to the call myself. Fearing I may become a drone to the Association I would like to withdraw from it, and herewith I present my resignation and also express my strong desire for its welfare."]

Drs. A. T. Barr, G. W. Worth, S. A. Gerry and O. M.

Carleton being present, were duly elected members of the Association.

The Association then proceeded to the election of officers for the ensuing year, which resulted as follows:

Dr. Joseph Mansfield, *President;* Dr. C. B. Porter, *Vice President;* Dr. A. T. Metcalf, *Recording Secretary;* Dr. S. C. Whiting, *Corresponding Secretary*; Dr. H. Benedict, *Treasurer.*

The unfinished business of last meeting was then taken up.

Dr. Foster, chairman of committee on "a general Fee Bill for the Dentists of the State," reported the list of prices as adopted by committee, which, on motion of Dr. Porter, was then taken up by article and amended.

[As the fee for extracting was the only one that elicited much discussion, I omit the rest.]

Dr. Whiting said that half a dollar was little enough for extracting a tooth. He formerly charged but a quarter, but since he charged a half, he found that his patients paid it just as freely, and left his office much better satisfied with the operation.

Where a person is too poor to pay a half dollar for having a tooth extracted, thinks best to charge nothing. Expresses a desire that all the Dentists present would make a half dollar their price for extracting, and felt confident in saying that, after one year's practice at that, they would never fall back on the old two shilling rule.

Dr. Metcalf said that the greatest objection he had to charging half a dollar for such a simple operation was, that people would judge from it that all our fees were in proportion. Had no particular objection in trying it till the next meeting, but thought best to *elevate* on other operations.

Dr. Foster said that in every State in the Union, except Michigan, Dentists charged half a dollar for extracting, and in the large cities, the usual price is one dollar. In Chicago, not two hundred miles distant, the regular price is a dollar. Thought the Dentists of Michigan ought not to be so far behind the rest of the world as to be getting only twenty-five cents for extracting.

Dr. Bancroft remarked that in a country town like his, where physicians were glad to get chances to extract teeth for any price they could get, he thought it would be rather difficult to get a half dollar, but was willing to try it.

Dr. Porter said that the people of Ann Arbor thought two shillings were quite enough to pay for extracting a tooth; but he was in favor of half a dollar.

With him, one or two napkins were always soiled in the operation, and two shillings would not much more than pay for washing them.

The yeas and nays were called for, which resulted in favor of making half a dollar the regular fee for extracting.

Considerable discussion arose as to the practicability of a general fee bill for the Dentists of the State, the principle argument of which was, that Dentists in the country could afford their services at a less rate than those in the city, as they were not subjected to near the expense. A vote was afterward taken, which showed a unanimous expression in favor of a general fee bill.

On motion of Dr. Foster, the two articles appended to the Fee Bill, as presented by committee, were ordered to be printed with it.

On motion, Drs. Porter, Metcalf and Foster, were appointed a committee on printing fee bill.

On motion of Dr. Porter, Drs. Whiting, Bancroft and Gerry, were appointed a committee to draft and report the order of business for the present session. Adjourned.

AFTERNOON SESSION.

Members met promptly at 1½ o'clock, and on invitation of Dr. Porter, proceed on a visit to the State Medical College and University. After a very pleasant and interesting visit of about two hours, the members returned, considerably refreshed in "body, mind and *spirits*."

At $3\frac{1}{2}$ o'clock, the Association was called to order by the President.

Dr. Whiting, chairman of committee on order of business, reported the following:

1st, President's address; 2nd, Vice President's address; 3d, dissertation on fang filling, by Dr. Metcalf.

ORDER OF DISCUSSION.

1st, Preparation of Cavities; 2nd, Filling Teeth; 3d, Fang Filling; 4th, treatment of Exposed Nerves; 5th, method of destroying Nerve for Fang Filling; 6th, Alveolar Abscess, cause and cure; 7th, taking impressions; 8th, Gold Solder; 9th, Best metal for casts, and method of making.

After several calls for the first address, the President said:

"Gentlemen of the convention, I should be gratified no less than yourselves, to have appeared before you with an address suited to the occasion. As it is, I can only thank you for the honor you have conferred upon me by reëlecting me to the office of President. As I do not feel competent to serve you in this capacity, I can do so only to the best of my ability, and hope for your assistance in performing the duties of my office. I must express to you the sincere gratification it is to me, that so many members of our profession have responded to our call. We have long needed an Association of this kind. We meet for the mutual elevation and improvement in the science of Dentistry. It is a duty we owe to our profession, our patrons and ourselves, to thoroughly understand every branch of our art. We are differently situated from the Dentists in large cities. It is not only necessary for us to understand mechanical Dentistry, but we must be familiar in the treatment of diseases of the teeth, and the best method of conducting second dentition.

This is our fourth meeting; and though we do not boast of a great amount of talking talent, who of us that have attended the meetings, can say that he has not been benefited by them. We hope to make it still more instructive and beneficial to its members than it has been, so that persons receiving our service, shall receive as skillful treatment as our department in science can confer. It is the *duty* of every member to do all in his power to elevate the platform of his profession. Let us, one and all, add our mite of knowledge to the general fund, and with an unyielding determination, find a place in the front rank of operators."

The Vice President being called upon, said he was not a talking man, and not having expected to be called on for an address, he would beg to be excused. He welcomed the members of the profession to Ann Arbor; and felt encouraged at seeing so many present. Hoped that other Dentists in the State would see the benefit of such an Association, and assist in carrying on the work that has been so generously begun.

Dr. Metcalf being then called upon said: At the last meeting of the Association he was invited to prepare a paper on fang filling to be read at this time. He thanked the Association for the compliment paid him by the invitation, and regretted that other duties had prevented. As the subject of fang filling was in the order of discussion, he would defer what he might have to say on the subject till it comes up in the regular order of discussion.

Dr. Foster being called upon to make some remarks said: He was in the same fix as Dr. Porter. Thanked the members for calling on him to speak. Gave an interesting description of a tour among the Eastern and Southern Dentists, during the past summer.

On motion, the Association proceeded to take up the order of discussion.

1ST, PREPARATION OF CAVITIES.

Dr. Whiting being called for, said: He did not know as he could say any thing new on this subject. His rule is to make the orifice as large as the cavity, where it can be done without encroaching too much upon the enamel.

Dr. Gerry said he liked a large cavity in order to fill to advantage, and for that reason, if no other, he always makes the orifice full as large as the cavity, if possible to do so without impairing the strength of the tooth. If he could excavate a cavity, he could fill it.

Dr. Foster does the principle part of excavating and shaping small cavities with a drill.

On motion, it was concluded to discuss the first and second articles in the order of discussion, at the same time.

Dr. Barr said he could explain his ideas better by doing the work than by telling his experience. Made some remarks on his manner of preparing and filling cavities. Prepares his foil in strips or ribbands, and folds them regularly and evenly into the cavity. Always sure to have each fold project a little out of the cavity.

Dr. Metcalf said he believed that was the old way as recommended and taught by Dr. Harris. Did not doubt but that an excellent filling could be made in that way, but thought we had two or three better modes of filling. Believed gold in the form of cylinders far preferable to the strips, and felt confident in pronouncing the Chrystal Gold Foil in the hands of patient and skillful operators, the very best material extant. There could not be a doubt of it, in his opinion. On account of securing a firm starting point (which is a necessary requisite to a good filling of the Chrystal Gold Foil) he did not use it much in approximal cavities.

Dr. Porter said that in cleaning cavities he adopted about the same plan as mentioned by Drs. Gerry and Barr, but used cylinders mostly in filling. Described a case of the second superior bicuspid, where there was nothing left of the crown but a buccal and palatial projection of enamel, which he filled with cylinders and chrystal gold foil.

Dr. Metcalf inquired if there were any teeth back of the bicuspids?

Dr. Porter. There was a second molar.

Dr. Metcalf. Would it not have been easier or better to have secured a smooth plate of some kind, on the posterior aproximal surface of the tooth, by means of a wedge between it and the molar, and then filled entirely with chrystal gold foil.

Dr. Porter, did not believe he could have filled it in a more satisfactory manner.

Dr. Carleton gave his method of preparing cavities where the nerve is very nearly exposed. Uses a varnish to cover the tender spot.

Dr. North finds most trouble in filling the approximal cavities of incisors. Where the nerve is slightly exposed, uses a little gum arabic to hold a small block or cap of gold over the nerve, while he is introducing the filling. Uses his gold in strips somewhat narrower than is usual, and thinks he has better success with it.

Dr. Mansfield remarked that our success in arresting decayed teeth, depended much in properly excavating the cavities. For grinding surfaces, uses gold in the form of pellets.

Dr. Gerry, would like to inquire the use of gums, as mentioned by Drs. Carleton and North. He considered such things decidedly injurious to the teeth.

[Rather a warm discussion arose as to the use of gums over exposed nerve, but it was just twilight and the discussion was not written out.]

Dr. Gerry suggested taking up in this connection, the materials for filling teeth.

Dr. Whiting objected: said that the subject of fang filling would be more interesting.

On motion of Dr. Foster, Association adjourned for one hour.

EVENING SESSION.

Association was called to order at 7 o'clock, President Mansfield in the chair.

Dr. Whiting made a motion that the Association proceed to the discussion of the fourth article, viz. "Fang Filling."

Dr. Whiting was called for. Said he had seldom tried double teeth, but had confined his operations in fang filling to those teeth that have but one nerve. Has seldom tried the operation on teeth that he found dead. Prefers destroying the nerve himself, and when the patient will submit to it, prefers to do so with an instrument, and usually fills immediately. Occasionally finds a case where bleeding will not admit filling quite so speedily ; but usually strong camphor will suppress the bleeding, in which case he proceeds with filling. For destroying nerve, uses spiral spring wire (gold) filed to the proper shape and barbed. Has had but one bad case where he has destroyed the nerve with an instrument.

Dr. Metcalf has done a little at fang filling, and so far has been successful in every attempt. Proceeds with great caution, and never fills the fangs and crown at one sitting. Neither would attempt destroying the nerve with an instrument, and filling the root at the same sitting. Has treated between thirty and forty cases within the past four months. Seldom destroys the nerve with an instrument, and always treats the nerve cavity with creosote previous to filling. Many of his operations were on fangs that had been ulcerated, some of them for years, and had formed a fistulous opening through the gum, for the discharge of matter. He related three cases with which he had had some difficulty, but which were all right at last. Thinks the operation of fang filling deserves the especial attention of dentists. Thought our facilities for the construction of artificial dentures, stimulated us in the too free use of the forcep. Hoped the dentists present would pay more attention to this portion of their practice, and each one be able to report a long list of cases at the next meeting. Could not advise them to fill every old *snag* that may be presented, but whenever they found a dead tooth in a healthy mouth, and the adjoining teeth in a good condition, be sure and make a trial upon it.

Dr. Bancroft remarked that he exerted himself more to save the nerve, if exposed, than he does to save a dead fang. Has treated the fangs of some teeth that he found dead with good success.

Dr. Porter said that he has had good success in fang filling, even with those that had been ulcerated for a long time. Treats mostly with nitrate of silver. Related a case that appeared to him at first, an obstinate one—a horridly ulcerated old root—but which yielded to treatment in a few days, was thoroughly cleansed and filled, and gave no further trouble. He treated the case with chloride of potassium. Another case that had been ulcerated for two or three years and constantly discharging matter, he treated in a similar manner and with equal success.

Dr. Gerry has often filled the fangs of incisors, but only in cases where he found the nerve already destroyed. His operations so far have been successful.

Dr. Metcalf thinks such cases more difficult than where the nerve is purposely destroyed for the operation.

Dr. Porter inquired if any present had filled fangs with plaster Paris and covered with gold.

Dr. Metcalf hoped there was no one present had attempted such a thing. Related a severe case of alveolar abscess caused by a lower molar, and wished to get the sense of the members present on treatment.

Dr. Mansfield related a similar case.

Dr. Bancroft motioned to take up the subject of taking impressions.

President Mansfield said that would be in order if there was nothing more to be said on the subject of fang filling.

7. *Taking Impressions.*—Dr. Whiting was called up. Said he could say nothing new on this subject. He always uses plaster Paris where there are no teeth to interfere with a good impression. Does not use salt in the plaster, on account of taste. Uses wax only where remaining teeth prevent from drawing off a plaster impression.

Dr. Gerry said that he takes impressions in plaster in nearly every case, and would give his manner of using it, as it might be new to some present. He prepares the mouth first, by building wax nicely around the necks of the teeth, and then proceeds with plaster the same as if there were no teeth. Thinks a more perfect impression can be had of the necks of the teeth, in this way, than in any other manner he is acquainted with.

Dr. Porter takes impressions of both upper and lower jaw with plaster Paris, except in cases of partial sets, where he uses wax.

President Mansfield remarked that it was about time to adjourn, and as there was some business to transact, the discussion better be closed.

On motion, the discussion was closed.

Treasurer's report was called for, read and approved. On motion warrants were ordered drawn on the Treasurer to pay bills in the hands of the Secretary, amounting to $8,50.

Dr. Porter motioned that the fee bill take effect on the first day of April next, which was carried.

The Secretary then read a resolution of thanks to the professors of the Medical College and University, for the many courtesies shown the members of the "Michigan Dental Association" while visiting their institutions, which was adopted.

On motion the Secretary was instructed to draft a copy of the proceedings of this meeting, and forward to Messrs. Taft and Watt, for their disposal.

On motion the thanks of the association were tendered to Dr. Porter for opening his office to the use of the Association, and for many attentions during the convention.

On motion, adjourned to meet at the office of Drs. Whiting & Benedict, in the city of Detroit, on the second Tuesday of January, 1859.

